# Adaptation and application of the Parent Attitudes About Childhood Vaccines survey tool in the Vietnamese language: a cross-sectional study

**DOI:** 10.1186/s12889-024-18389-x

**Published:** 2024-04-02

**Authors:** Bao Quy Quoc Truong, Ken Ing Cherng Ong, Akira Shibanuma, Junko Kiriya, Masamine Jimba

**Affiliations:** 1 Department of Community and Global Health, Graduate School of Medicine, The University of Tokyo, Tokyo, Japan; 2 Institute for Community Health Research, University of Medicine and Pharmacy, Hue University, Hue, Vietnam; 3 Department of Epidemiology, Harvard T.H. Chan School of Public Health, Boston, MA USA

**Keywords:** Vaccination hesitancy, Vaccines, Parents, Child, Immunisation programs, Vietnam

## Abstract

**Background:**

Parental vaccine hesitancy could lead to outbreaks of vaccine-preventable diseases. Although parental vaccine hesitancy exists in the Vietnamese community, no research has directly investigated this social phenomenon in Vietnam. Among the validated measures, the 15-item Parent Attitudes About Childhood Vaccines survey tool (PACV) was reliable for predicting vaccine-hesitant parents. However, the PACV was not available in Vietnamese. This study aimed to develop a Vietnamese version of the PACV and examine factors associated with parental vaccine hesitancy in Hue city, Vietnam.

**Methods:**

This study was a cross-sectional study. The English PACV was translated into Vietnamese with content and face validation. Self-administered questionnaires were distributed to 400 parents at ten commune health centres in Hue city, Vietnam. The parents were asked to answer the questionnaire again after two weeks for the test–retest reliability. The Vietnamese PACV reliability was assessed using Cronbach’s alpha and McDonald’s omega, and the intra-class correlation (ICC) coefficients were used for the test–retest reliability. The construct validity was tested by the hypothesis that parental vaccine hesitancy would be related to the intention of getting the children vaccinated. Exploratory factor analysis was also undertaken to determine the construct validity. Bivariate and multivariable logistic regression were used to identify the factors associated with parental vaccine hesitancy.

**Results:**

The Vietnamese PACV final version (PACV-Viet) contained 14 items. Three hundred and fifteen parents returned completed questionnaires, giving a response rate of 78.8%. The Cronbach’s alpha and McDonald’s omega were 0.72 and 0.70, respectively. Out of 315 parents, 84 responses were returned for test–retest reliability. All ICCs were good to excellent, ranging from 0.81 to 0.99. The PACV-Viet was confirmed to have construct validity. Using the PACV-Viet, 8.9% of the parents were found hesitant to childhood vaccination. Being unemployed and having seen the news about adverse events following immunisation were associated with parental vaccine hesitancy, with AOR = 3.2 (95% CI 1.3–8.0) and AOR = 4.5 (95% CI 1.2–16.7), respectively.

**Conclusions:**

The PACV-Viet is a valid and reliable tool. Community outreach is necessary to alleviate parents’ concerns about childhood vaccination.

## Background

Vaccines have significantly contributed to global health by reducing the burden of numerous infectious diseases [1]. Despite the importance of vaccinations, many parents have been hesitant to some or all vaccines for their children [2]. According to the Strategic Advisory Group of Experts on immunisation of the World Health Organisation, vaccine hesitancy is defined as ‘delay in acceptance or refusal of vaccines despite availability of vaccination services’ [3]. This concept encompasses a spectrum of attitudes and behaviours, from actively demanding vaccines to completely denying all vaccines [4]. The delay or refusal might cause under-immunisation and reduce the population’s protection through weakening herd immunity. Parental vaccine hesitancy could lead to outbreaks of vaccine-preventable diseases [5].

Parental vaccine hesitancy is not a novel phenomenon [6]. However, national assessments could have been more comprehensive for intervention to reduce hesitancy globally [2, 7]. Besides, few studies were conducted on parental vaccine hesitancy in South-East Asian countries. In Malaysia, 11.6% of parents have hesitated about their children’s vaccination [8]. In Indonesia, 15.9% of the parents were hesitant [9]. In the Philippines, the rate was 36.4% among urban respondents [10]. In Vietnam, parents’ concerns have arisen after the vaccine’s post-injection reactions were reported [11]. However, parental vaccine hesitancy has never been studied in the country. Vaccine hesitancy in one region could have far-reaching repercussions elsewhere due to the transmission of diseases and disinformation. This emphasises the need for global surveillance of parental vaccine hesitancy.

Among the validated tools for parental vaccine hesitancy measure, the Parent Attitudes about Childhood Vaccines (PACV) is one of the most frequently used [12]. Initially developed in the English language in the United States, the tool is divided into three domains ‘Safety and efficacy’, ‘General attitudes and trust’ and ‘Behaviour’ [13, 14]. The PACV is a robust tool to reliably identify vaccine-hesitant parents [15]. The PACV validation will allow more robust studies on parental vaccine hesitancy. As this tool has not yet been validated in Vietnamese, its validation would help create a tool that could identify parental vaccine hesitancy in Vietnam.

Therefore, it is important to adapt the PACV, conduct its validation study in the Vietnamese language, and subsequently identify factors associated with parental vaccine hesitancy in Vietnam. This study was conducted to (i) develop a Vietnamese version of the PACV and (ii) examine factors associated with parental vaccine hesitancy in Hue city, Vietnam.

## Methods

### Study design and settings

This was a cross-sectional study. In 2021, Hue city had 27 wards, each with a commune health centre. These are the primary points of vaccine delivery in the Vietnamese Expanded Programs on Immunisation (EPI) [16]. The study assigned every centre a number. Then, the random number function (RAND) was used to generate random numbers in Microsoft Excel. Ten matched centres were chosen for the data collection. The selected wards' population accounted for 46% of the total population of Hue city [17]. A research assistant was assigned to each chosen centre to help with the recruitment and data collection. The research assistants were students and staff from the Faculty of Public Health, Hue University of Medicine and Pharmacy in Hue city, Vietnam.

### Participants and inclusion criteria

The study collected data from parents. Inclusion criteria included (i) mothers and fathers aged 18 and above, (ii) having at least a child aged less than five years old. Parents were excluded from the study if they did not wish to participate or had been living in Hue city for less than three months.

### Translation of the PACV into Vietnamese

The English PACV was translated into Vietnamese language using the back translation method [18]. First, two forward translations were done by two bilingual translators. Then, the researcher (BQQT) synthesised the translations with revisions from the translators to create one common translation. Another bilingual translator translated this common translation back into English. The inconsistencies were identified and modified to ensure an accurate translation. After revision, the common translation was reviewed again by an expert panel for content validity. The panel included three experts: a paediatrician, a public health academic, and a senior researcher. The last stage was the pre-test. The pre-final version was distributed to 30 parents from the target criteria. These parents shared the same inclusion criteria but were not included in the primary survey. Following the pre-test, each parent provided feedback to determine which scale items were comprehensible and difficult to understand. Then, the Vietnamese PACV was finalised with achieved face validity.

### Sample size estimation

The sample size was estimated using the formula for detecting a difference between two proportions [19]:

$${\text{n}}={\left({Z}_{\frac{\alpha }{2}}+{Z}_{\beta }\right)}^{2}\left(\frac{{p}_{1}\left(1-{p}_{1}\right)+{p}_{2}\left(1-{p}_{2}\right)}{{\left({p}_{1}-{p}_{2}\right)}^{2}}\right)$$

Due to the lack of data on vaccine hesitancy in Vietnam, the calculation used published data from another South-East Asian country. According to a study in Malaysia using the PACV, 20.4% of vaccine-hesitant parents were under 30, while 8.4% were aged 30 or older [8]. For a confidence level of 95%, α is 0.05, and 1.96 is the critical value Z_α/2_. Supposing a power of 80%, 0.84 is the critical value of the normal distribution Z_β_ at β of 0.2. Using the above formula, 131 was the minimum required sample size (n) for each age group to detect the stated difference between the two proportions. Considering 10% of sample loss, 288 was the study’s minimum sample size. However, for scale validation studies, it is recommended a sample size of 300–450 participants [20, 21]. Thus, the study aimed to recruit 400 parents as a feasible final sample size.

### Parents' recruitment

The parents were recruited at commune health centres from September 10 to October 15, 2021. Each centre usually had one or two EPI sessions per month. The parents were recruited during or after their child’s EPI session. A child’s mother and father could both participate in the study. The recruited parents might not necessarily be a couple. Either or both parents received the information sheet and signed the informed consent form. In Vietnamese society, mothers’ roles were traditionally responsible for the children’s health [22]. Thus, more mothers accompanied their children on the immunisation day than fathers. The parents’ recruitment ended when the sample size reached 400.

### Data collection

The data were collected using a paper-based questionnaire, which the parents completed at the commune health centres. The questionnaire included the Vietnamese PACV and other questions such as parents’ gender, parental educational level and employment status, number of children [8, 9, 23]; information sources on vaccination, hearing about the adverse events following immunisation [24]; and other socio-demographic characteristics [25]. The responses to the questionnaire were collected after the parents finished. With consent, the parents were asked to answer the Vietnamese PACV again after two weeks for the test–retest reliability [26]. In the second test, the Vietnamese PACV was sent to all participating parents using Google Forms via email or mobile short messages.

### The PACV scoring

The PACV was a self-administered tool. It contains 15 items divided into three-factor domains: ‘Behaviour’ (items Q1 and Q2), ‘Safety and efficacy’ (items Q7–Q10) and ‘General attitude and trust’ (items Q3–Q6 and Q11–Q15) [13]. Items Q1, Q2 and Q11 had the answering options yes, no and do not know. Items Q3 and Q15 had ratings from 0 to 10. Items Q4-Q7 and Q13-Q14 had the answering options including strongly agree, agree, not sure, disagree and strongly disagree. Items Q8-Q11 had the answering options including not at all concerned, not too concerned, not sure, somewhat concerned and very concerned. Item Q12 had the answering options including not at all hesitant, not too hesitant, not sure, somewhat hesitant and very hesitant. Hesitant responses were assigned a 2, ‘do not know or not sure’ a 1, and non-hesitant responses a 0. The ‘do not know’ responses in items Q1 and Q2 were excluded as missing data. Based on the original validation study, the total raw score was converted to a 0–100 scale using a linear transformation. The converted scores were dichotomised into two categories followed by the developer: non-hesitant (score < 50) and hesitant (score ≥ 50).

### Data analysis

The study used IBM’s SPSS version 26 for data management and Stata software (Stata/SE 16.1, College Station, Texas) for statistical analyses. Items Q1 and Q2 were not included in the reliability and construct validity analysis because they are dichotomous, unlike other Likert questions of the PACV [21]. Negative items were reverse-coded. All psychometric tests used standardised items [27]. The study carried out an initial Cronbach’s alpha analysis and principal component analysis (PCA) without rotation and forcing one component. Then, the Vietnamese PACV’s items were checked if they could be removed from the survey tool, based on corrected item-total correlation values, Cronbach’s alpha if the item was deleted, and factor loadings of the PCA [28, 29]. Descriptive statistics were used for demographic data, parents’ responses and the Vietnamese PACV’s scoring. A Fisher's Exact test was used for hypothesis testing.

The Vietnamese PACV reliability was evaluated using Cronbach’s alpha and McDonald’s omega. Cronbach’s alpha and McDonald’s omega of 0.7 are acceptable for internal consistency [21]. Test–retest data were assessed by calculating the intra-class correlation coefficients (ICCs). The ICC analysis used a two-way mixed-effects model, absolute agreement and average measurement (ICC(3,1)). An ICC of 0.6 is considered acceptable [30].

To determine the construct validity, the study assessed its convergent aspect [21]. However, there were limited validated measures available in Vietnam. Hence, the study hypothesised that parental vaccine hesitancy would be related to the intention of getting the children vaccinated. The positive correlation might indicate that the scale has construct validity [31]. Exploratory factor analysis (EFA) was done to determine the construct validity further. The study used Bartlett’s sphericity test to determine data appropriateness and the Kaiser-Meyer Olkin (KMO) to determine sampling adequacy. An applicable factor analysis is indicated with a minimum KMO value of 0.5 and a significant Bartlett’s sphericity test. The study assessed the dimensionality using Promax rotation. The number of factors was shown by the scree plot [32].

Bivariate and multivariable logistic regression were used to examine the factors associated with parental vaccine hesitancy. Variance inflation factor (VIF) was used for the multicollinearity test. VIF greater than 5 is a sign of detecting multicollinearity [33]. The significant level was set at 0.05.

## Results

### The Vietnamese version of the PACV

The expert panel agreed that the translation was appropriate for the Vietnamese population and the given purpose, but suggested minor improvements for precision. Item Q3 (‘How sure are you that following the recommended shot schedule is a good idea for your child?’) measured the scope to which the parent believes that having a vaccination is a good idea. This question response was based on a 0–10 scale. However, the Vietnamese meaning (‘How sure are you that …’) referred to a yes/no question and could confuse the respondent. An appropriate version of item Q3 was recommended by adding ‘To what extent …’ to the Vietnamese question. Item Q4 (‘Children get more shots than are good for them’) was noted to be difficult to interpret. Although one expert considered item Q4 was not grammatically correct when translated to Vietnamese, the panel decided to keep the literalness of the question. In addition, items Q14 (‘I am able to openly discuss my concerns about shots with my child's doctor’) and Q15 (‘All things considered, how much do you trust your child's doctor?’) also needed to be slightly modified by changing ‘child's doctor’ to ‘vaccination consulting doctor’, because children often do not have their own doctor in Vietnam.

Following the content validation by the expert panel, the pre-final Vietnamese PACV was produced and ready for face validity testing. Among 30 parents who participated in the pre-test, feedback was collected with no significant complaint, and no question was considered difficult to understand. Almost all the parents found the questionnaire easy to complete. The Vietnamese PACV was then used in the primary survey.

### Socio-demographic characteristics of the participants

Forty questionnaires were distributed to the parents in each of the ten commune health centres. In a total of 400 questionnaires, 315 were fully filled with information and returned, giving an estimated response rate of 78.8%. The parents' socio-demographic characteristics are outlined in Table 1. The majority of the parents were mothers (71.8%), and the mean age was 30.8 (SD 5.9) years. Around 68.3% of the parents were employed, and about two-thirds had seen information about adverse events following immunisation (70.8%).

**Table 1:** Socio-demographic characteristics of the participants

Characteristics	*N* = 315	%
Parent		
Mother	226	71.8
Father	89	28.3
Age		
18—29 years	132	41.9
≥30 years	183	58.1
Number of children		
1	96	30.5
2	163	51.8
3 and above	56	17.8
Ethnicity		
Kinh	313	99.4
Other	2	0.6
Religion		
None	160	50.8
Buddhism	139	44.1
Catholic	16	5.1
Marital status		
Married	312	99.1
Single	3	0.9
Education level		
Secondary school and below	82	26.0
High/Vocational/Technical school	91	28.9
College/University degree and above	142	45.1
Employment status		
Employed	215	68.3
Unemployed	100	31.8
Monthly household income in Vietnamese Dong (1 United States Dollar = 22,820 Vietnamese Dong as of December 2021)		
< 6,000,000	66	21.0
6,000,000—< 8,000,000	66	21.0
8,000,000—< 10,000,000	55	17.5
10,000,000 +	128	40.6
Preference on type of vaccine		
Free	268	85.1
Paid	47	14.9
Source of information on childhood vaccination		
No information/No source	7	2.2
Family	88	27.9
Friends and acquaintances	33	10.5
Healthcare professional	173	54.9
Public health authorities	212	67.3
Television	60	19.1
Print media	15	4.8
Internet searches	78	24.8
Social media networks	49	15.6
Have seen news about adverse events following immunisation		
Yes	223	70.8
No	92	29.2
Will take the COVID-19 vaccine		
Yes	268	85.1
No/Not sure	47	14.9
Will let the children take the COVID-19 vaccine (when possible)		
Yes	236	74.9
No/Not sure	79	25.1

*Abbreviations*: *N* number of parents, *COVID-19* Coronavirus Disease 2019

### The parents’ response and item removal

Table 2 provides descriptive statistics for the parent's response to the Vietnamese PACV. Approximately one-third (31.4%) of the 315 parents admitted to postponing their child’s shot for reasons other than illness or allergy, and 13.7% of them had decided not to vaccinate their child. Notably, parents were greatly concerned about the vaccines' side effects (79.1%). According to the response, some parents also considered themselves hesitant about childhood vaccination (15.9%). However, most parents agreed they could trust the information they received about the shots (93.3%) and the vaccination consulting doctor (87.3%).

**Table 2:** PACV-Viet statements and the parent’s response

Item		Response	Count (%)
Q1	Have you ever delayed having your child get a shot for reasons other than illness or allergy?	*Yes*	99 (31.4)
		No	210 (66.7)
		Don’t know	6 (1.9)
Q2	Have you ever decided not to have your child get a shot for reasons other than illness or allergy?	*Yes*	43 (13.7)
		No	259 (82.2)
		Don’t know	13 (4.1)
Q3	How sure are you that following the recommended shot schedule is a good idea for your child?	*0–5*	18 (5.7)
		6–7	19 (6.0)
		8–10	278 (88.3)
Q4	Children get more shots than are good for them	*Agree*	276 (87.6)
		Not sure	30 (9.5)
		Disagree	9 (2.9)
Q5	I believe that many of the illnesses that shots prevent are severe	*Disagree*	9 (2.9)
		Not sure	32 (10.2)
		Agree	274 (87.0)
Q6	It is better for my child to develop immunity by getting sick than to get a shot	*Agree*	31 (9.8)
		Not sure	48 (15.2)
		Disagree	236 (74.9)
Q7	It is better for children to get fewer vaccines at the same time	*Agree*	101 (32.1)
		Not sure	116 (36.8)
		Disagree	98 (31.1)
Q8	How concerned are you that your child might have a serious side effect from a shot?	*Concerned*	249 (79.1)
		Not sure	18 (5.7)
		Not concerned	48 (15.2)
Q9	How concerned are you that any one of the childhood shots might not be safe?	*Concerned*	220 (69.8)
		Not sure	30 (9.5)
		Not concerned	65 (20.6)
Q10	How concerned are you that a shot might not prevent the disease?	*Concerned*	183 (58.1)
		Not sure	58 (18.4)
		Not concerned	74 (23.5)
Q11	If you had another infant today, would you want him/her to get all the recommended shots?	*No*	2 (0.6)
		I don’t know	11 (3.5)
		Yes	302 (95.9)
Q12	Overall, how hesitant about childhood shots would you consider yourself to be?	*Hesitant*	50 (15.9)
		Not sure	38 (12.1)
		Not hesitant	227 (72.1)
Q13	I trust the information I receive about shots	*Disagree*	2 (0.6)
		Not sure	19 (6.0)
		Agree	294 (93.3)
Q14	I am able to openly discuss my concerns about shots with my child’s doctor	*Disagree*	4 (1.3)
		Not sure	16 (5.1)
		Agree	295 (93.7)
Q15	All things considered, how much do you trust your child’s doctor?	*0–5*	15 (4.8)
		6–7	25 (7.9)
		8–10	275 (87.3)

*Italic* answers indicate hesitancy

*Abbreviation*: *PACV-Viet* The Vietnamese version of the Parent Attitudes About Childhood Vaccines survey tool

Table 3 shows the mean, SD, corrected item-total correlation for each item, alpha value if the item was deleted and the factor loadings on the PCA. The corrected item-total correlation was negative for item Q4. Removing item Q4 could increase the alpha. Moreover, item Q4 also had a negative factor loading. Therefore, item Q4 was deleted and omitted from further analysis. As a result, the Vietnamese version of the PACV (PACV-Viet) contains 14 items.

**Table 3:** The PACV-Viet items with Cronbach’s alpha if item deleted and PCA factor loadings

Items		Mean	SD	Corrected item-total correlation	Alpha (If Item Deleted)	Loadings
Q3	How sure are you that following the recommended shot schedule is a good idea for your child?	9.1	1.5	0.5	0.59	0.4
Q4	Children get more shots than are good for them	1.7	0.8	-0.4	0.72	-0.3
Q5	I believe that many of the illnesses that shots prevent are severe	1.8	0.7	0.2	0.64	0.3
Q6	It is better for my child to develop immunity by getting sick than to get a shot	3.8	0.9	0.2	0.63	0.2
Q7	It is better for children to get fewer vaccines at the same time	3.0	1.0	0.2	0.64	0.1
Q8	How concerned are you that your child might have a serious side effect from a shot?	3.9	1.1	0.3	0.62	0.2
Q9	How concerned are you that any one of the childhood shots might not be safe?	3.8	1.2	0.4	0.61	0.2
Q10	How concerned are you that a shot might not prevent the disease?	3.6	1.2	0.2	0.63	0.2
Q11	If you had another infant today, would you want him/her to get all the recommended shots?	1.1	0.2	0.2	0.63	0.2
Q12	Overall, how hesitant about childhood shots would you consider yourself to be?	2.0	1.1	0.5	0.59	0.3
Q13	I trust the information I receive about shots	1.7	0.6	0.5	0.58	0.4
Q14	I am able to openly discuss my concerns about shots with my child’s doctor	1.7	0.7	0.4	0.60	0.3
Q15	All things considered, how much do you trust your child’s doctor?	9.0	1.4	0.5	0.58	0.4

*Abbreviations*: *PACV-Viet* The Vietnamese version of the Parent Attitudes About Childhood Vaccines survey tool, *PCA* Principal component analysis, *SD* Standard deviation

### Reliability analysis

The overall Cronbach’s alpha and McDonald’s omega for the PACV-Viet were 0.72 and 0.70, respectively. Out of 315 parents, 84 questionnaires were returned from the retest after two-week intervals. As shown in Table 4, the ICC was good to excellent for each item, ranging from 0.81 to 0.99.

**Table 4:** Test–retest reliability of the PACV-Viet after two weeks

Items		ICC	95% CI
Q3	How sure are you that following the recommended shot schedule is a good idea for your child?	0.98	0.97—0.99
Q5	I believe that many of the illnesses that shots prevent are severe	0.85	0.77—0.90
Q6	It is better for my child to develop immunity by getting sick than to get a shot	0.89	0.84—0.93
Q7	It is better for children to get fewer vaccines at the same time	0.96	0.94—0.98
Q8	How concerned are you that your child might have a serious side effect from a shot?	0.81	0.71—0.88
Q9	How concerned are you that any one of the childhood shots might not be safe?	0.91	0.87—0.94
Q10	How concerned are you that a shot might not prevent the disease?	0.90	0.85—0.94
Q11	If you had another infant today, would you want him/her to get all the recommended shots?	0.89	0.82—0.93
Q12	Overall, how hesitant about childhood shots would you consider yourself to be?	0.91	0.85—0.94
Q13	I trust the information I receive about shots	0.86	0.79—0.91
Q14	I am able to openly discuss my concerns about shots with my child’s doctor	0.83	0.74—0.89
Q15	All things considered, how much do you trust your child’s doctor?	0.99	0.98—0.99

Number of test–retest questionnaires evaluated was 84

*Abbreviations*: *PACV-Viet* The Vietnamese version of the Parent Attitudes About Childhood Vaccines survey tool, *ICC* Intra-class correlation coefficient, *CI* Confidence interval

### Validity analysis

From the PACV-Viet, 28 (8.9%) parents were classified as vaccine-hesitant (score ≥ 50). As for the intention of getting the children vaccinated, 14.9% of the parents were not sure that they would keep their children fully vaccinated and on schedule in the future. Following the frequencies cross-tabulated in Table 5, a Fisher’s exact test was performed to determine the association between future vaccination intention with parental vaccine hesitancy. There was a significant association between the future vaccine intention and parental vaccine hesitancy (two-tailed *p*-value < 0.001).

**Table 5:** The association between hesitant parents and future vaccination intention

Hesitant parents	Future vaccination intention		Total
	Fully vaccinated and on schedule (%)	Not sure (%)	
No	261 (90.9)	26 (9.1)	287
Yes	7 (25.0)	21 (75.0)	28
Total	268 (85.1)	47 (14.9)	315
Fisher’s Exact Test			*P*-value <0.001

**Fig. 1:**
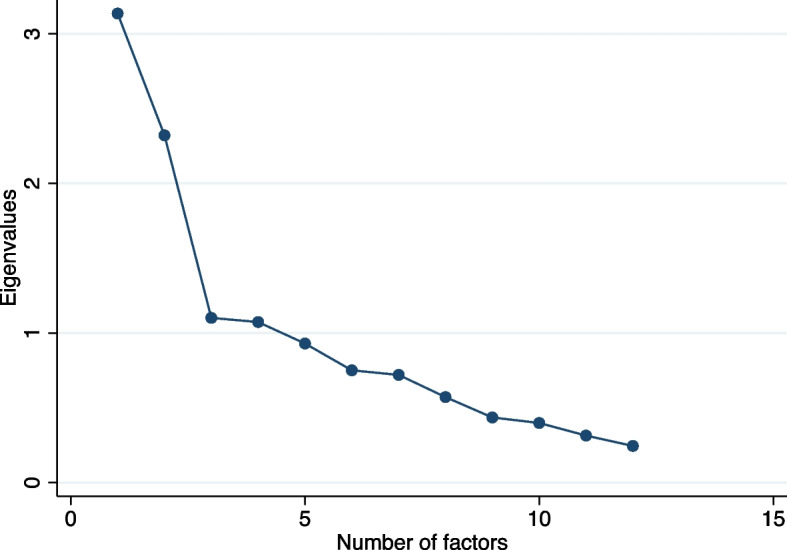
Scree plot of eigenvalues of factors in the PACV-Viet

The PACV-Viet were appropriate to proceed with factor analysis following the KMO value and Bartlett’s test of sphericity results. Using Promax rotation, the EFA identified four factors with Eigenvalues above 1, accounting for 63.6% of the total variance. On the scree plot, the curve’s elbow occurred at three (Fig. 1). Repeated testing with three-factor and four-factor models, three factors were the most conceptually suitable.

Table 6 shows the factor loading of items and three factors extracted from the EFA. Items Q3, Q5 and Q12-Q15 formed Factor 1, reflecting the ‘General attitudes’ domain from the original PACV. Items Q8-Q10 correlated to form Factor 2, the ‘Safety and efficacy’ domain from the original PACV. The remaining items Q6, Q7 and Q11 formed a new Factor 3, namely ‘Children and vaccination’.

**Table 6:** Items, factor loadings and factors of the PACV-Viet

		Factor 1	Factor 2	Factor 3
		General attitudes	Safety and efficacy	Children and vaccination
Q3	How sure are you that following the recommended shot schedule is a good idea for your child?	0.6		
Q5	I believe that many of the illnesses that shots prevent are severe	0.6		
Q12	Overall, how hesitant about childhood shots would you consider yourself to be?	0.4		
Q13	I trust the information I receive about shots	0.8		
Q14	I am able to openly discuss my concerns about shots with my child’s doctor	0.8		
Q15	All things considered, how much do you trust your child’s doctor?	0.6		
Q8	How concerned are you that your child might have a serious side effect from a shot?		0.8	
Q9	How concerned are you that any one of the childhood shots might not be safe?		0.9	
Q10	How concerned are you that a shot might not prevent the disease?		0.9	
Q6	It is better for my child to develop immunity by getting sick than to get a shot			0.5
Q7	It is better for children to get fewer vaccines at the same time			0.7
Q11	If you had another infant today, would you want him/her to get all the recommended shots?			0.5

*Abbreviation*: *PACV-Viet* The Vietnamese version of the Parent Attitudes About Childhood Vaccines survey tool

### Factors associated with parental vaccine hesitancy

Table 7 displays factors associated with parental vaccine hesitancy. The bivariate logistic regression model showed that being unemployed (OR = 2.7, 95% CI 1.3–6.0) and having seen the news about adverse events following immunisation (OR = 3.8, 95% CI 1.1–12.7) were associated with parental vaccine hesitancy. The results confirmed the significantly associated variables in the multivariable logistic regression model. The Hosmer–Lemeshow test indicated a good logistic regression model fit (*P*-value = 0.3), and the area under the curve was 0.78. The multicollinearity test resulted in a mean VIF of 1.59, indicating no serious multicollinearity problems in this model. When adjusted for all other variables, factors associated with parental vaccine hesitancy were being unemployed (AOR = 3.2, 95% CI 1.3–8.0) and having seen the news about adverse events following immunisation (AOR = 4.5, 95% CI 1.2–16.7).

**Table 7:** Factors associated with parental vaccine hesitancy

		Unadjusted		Adjusted	
Variables		OR (95% CI)	*P*-value	OR (95% CI)	*P*-value
Age (years)					
18—29 years	132	1		1	
from 30 years	183	1.3 (0.6—3.0)	0.488	0.7 (0.2—2.0)	0.493
Parent					
Father	89	1		1	
Mother	226	1.9 (0.7—5.2)	0.207	1.5 (0.5—4.4)	0.464
Number of children					
1	96	1		1	
2	163	1.3 (0.5—3.3)	0.595	1.5 (0.5—4.6)	0.483
3 and above	56	1.5 (0.5—4.8)	0.469	2.3 (0.6—9.3)	0.24
Religion					
None	160	1		1	
Buddhism	139	1.3 (0.6—2.8)	0.559	1.6 (0.7—4.0)	0.303
Catholic	16	0.8 (0.1—6.2)	0.792	0.8 (0.1—7.6)	0.879
Education level					
Secondary school and below	82	1		1	
High/Vocational/Technical school	91	2.4 (0.7—8.0)	0.151	3.2 (0.9—11.8)	0.086
College/University degree and above	142	1.6 (0.7—6.7)	0.195	2.5 (0.62 -9.8)	0.203
Employment status					
Employed	215	1		1	
Unemployed	100	2.7 (1.3—6.0)	0.012	3.2 (1.3—8.0)	0.014
Monthly household income in Vietnamese Dong (1 United States Dollar = 22,820 Vietnamese Dong as of December 2021)					
< 6,000,000	66	1		1	
6,000,000—< 8,000,000	66	0.4 (0.2—1.4)	0.129	0.3 (0.1—1.4)	0.121
8,000,000—< 10,000,000	55	1.4 (0.5—4.0)	0.505	1.5 (0.4—4.9)	0.542
10,000,000 +	128	0.5 (0.2—1.4)	0.166	0.5 (0.1—1.5)	0.2
Preference on type of vaccine					
Free	268	1		1	
Paid	47	2.1 (0.8—5.2)	0.123	3.0 (0.9—9.5)	0.067
Have seen news about adverse events following immunisation					
No	92	1		1	
Yes	223	3.8 (1.1—12.7)	0.034	4.5 (1.2—16.7)	0.027
Will take the COVID-19 vaccine					
Yes	268	1		1	
No or not sure	47	0.7 (0.2—2.3)	0.515	0.5 (0.1—2.0)	0.314
Will let the children take the COVID-19 vaccine (when possible)					
Yes	236	1		1	
No or not sure	79	1.8 (0.8—4.0)	0.178	2.2 (0.8—5.9)	0.112

*Abbreviations*: *OR* Odds ratio, *CI* Confidence interval, *COVID-19* Coronavirus Disease 2019

## Discussion

The study has two major findings. First, the most important finding was the development of a reliable and valid Vietnamese version of the PACV. To the best of the authors’ knowledge, the PACV-Viet is the first validated survey tool in Vietnamese for parental vaccine hesitancy. Second, the factors associated with parental vaccine hesitancy were being unemployed and having seen news about adverse events following immunisation.

During analysis, item Q4 was removed from the PACV-Viet, and this question also confused the translators and reviewers during the translation. This was likely because there was no precise translation from English to Vietnamese for ‘get more shots than are good’ without changing the author's expression. Since this study maintained the denotation of the question when making the PACV-Viet, most of the parents answered this item without getting the latent meaning. The problem was also noted in the development of the PACV and other validation studies [13, 34, 35]. This removal emphasises the importance of cultural adaptation in using survey tools.

In this study, items Q1 and Q2 were excluded from the psychometric properties testing. Dichotomous items require different reliability and validity assessment methods [21]. Besides, the ‘Don’t know’ response to these questions was for the parents who did not recall their child's vaccine record. This might be subjected to measurement errors [14]. In the Malay version, these items were also excluded from the score calculation for the same reason [35]. However, this study still counted these items. It could be a signal of parental vaccine hesitancy if delays or cancellations of vaccination were not medically justified [36].

The PACV-Viet had acceptable overall Cronbach's alpha and McDonald's omega, which were 0.72 and 0.70, respectively. With good to excellent values of the ICC, the PACV-Viet is stable and reliable over time. The PACV-Viet was also confirmed to have convergent validity through hypothesis testing. Similar to the finding, the PACV's outcomes were associated with future child immunisation status or odds of the non-timeliness of the first dose of measles [15, 37]. According to a study in Canada, higher PACV scores were associated with greater uncertainty about the intention to vaccinate children [31]. Although the predictive validity had not yet been confirmed in this study, the result suggests the PACV-Viet could be used as an intervention tool for early predicting parental vaccine hesitancy.

In the PACV-Viet, the EFA yielded a three-factor model. However, there were differences in the factor-loading structure of items Q6, Q7 and Q11. These questions both mentioned how the parents were concerned about getting their children vaccinated. Thus, they formed a new domain labelled ‘Children and vaccination’. The Malay version also bore a different factor structure [35]. On the other hand, a Turkish validation study confirmed the domain's structure of the original PACV using confirmatory factor analysis [38]. These differences are somehow expected, especially in different cultural adaptations of survey tools.

Using the PACV-Viet, 8.9% of the parents were vaccine-hesitant in Hue city, Vietnam. This finding is comparable with recent similar studies using the PACV, such as results from Peru (9.8%), Iraq (9.9%), Saudi Arabia (11%), Malaysia (11.6%) and the United Arab Emirates (12%). The finding is lower in this study than those in other studies, including Ireland (15%), Canada (15%), Indonesia (15.9%), Italy (34.7%) and some in the United States (> 20%). These differences are likely due to the characteristics of the studied populations and settings. With a hesitancy rate of 8.9%, however, one-third of the parents reported they had delayed their children’s vaccination. According to a study about the timeliness of vaccination, only 33% of the parents had their children vaccinated on schedule in Hue city, Vietnam [39]. Besides, the hesitancy rate is lower than the self-reported rate of parents who thought they could be vaccine-hesitant. This suggests that a substantial number of parents were accepting to vaccinate their children but were still concerned about the vaccines.

Being unemployed was significantly associated with parental vaccine hesitancy. Compared with other studies in the South-East Asia region, the result was consistent with the Malaysian study [8]. The data collection period coincided with the COVID-19 pandemic. Thus, the study noticed a high rate of unemployed parents [40]. Indeed, unemployed people were more likely to have negative feelings about vaccine safety, especially those with a low level of education and low household income [41]. In the United States, unemployed people had poorer influenza vaccine uptake rates and COVID-19 vaccination acceptability [42]. With the combined impact of COVID-19 and unemployment, parents might hesitate to vaccinate their children due to their limited resources, such as time and income. The unemployed parents might be more disadvantaged and susceptible to vaccine-hesitant attitudes, influencing the vaccination decisions [43].

Parents were more likely to be vaccine-hesitant if they had seen the news about adverse events following immunisation. In a study in northern Vietnam, many urban participants would refuse vaccination after hearing news about adverse events following immunisation in the media [24]. In another study in Danang, the media significantly affected Vietnamese mothers’ decision to have their daughters receive the human papillomavirus vaccine [44]. However, this association might also be due to reverse causality [45]. The vaccine-hesitant parents might tend to seek out or put more awareness on the news about adverse events following immunisation. They might also selectively register information supporting vaccine-hesitant viewpoints because it allows them to see what they want to see, i.e., confirmation bias [46]. Thus, while access to information is essential, some information also creates concerns, exacerbating mistrust and confusion about vaccination.

The study has some limitations. First, the representativeness of data could be limited since the recruitment was done at commune health centres. The study also did not account for commonalities among parents within selected centres. Thus, the clustering effect could not be investigated. Second, self-administered questionnaires might raise the social-desirability bias as the participants might not well remember the information and answer questions to their advantage. Moreover, parental vaccine hesitancy might be a sensitive topic to some parents, which could not accurately reflect the actual condition. The parents might already consider themselves vaccine-hesitant and refuse to give accurate answers. Third, the study used a categorical variable (i.e. the intention of getting the children vaccinated) instead of a continuous variable (i.e. another scale) in the construct validity analysis. A categorical variable is less informative and can be more difficult to compare to other scales of the same construct. Besides, correlational methods are commonly used to assess convergent validity, which is more accurate when the variables are continuous [47, 48]. The study also did not include parallel analysis for factor analysis, which could have provided additional insights into factor modelling. Last but not least, the research was limited to Hue city, Vietnam, and the findings might not reflect the entire country's prevalence of parental vaccine hesitancy.

## Conclusions

In conclusion, this study developed the PACV-Viet using several validation processes. Notably, the 14-item PACV-Viet was found reliable and valid. The tool can be used to report parental vaccine hesitancy among the Vietnamese population. It is valuable to identify parental vaccine hesitancy in Hue City, Vietnam. The findings could contribute significantly to local and regional knowledge on this important topic. Community-based outreach can be instrumental in addressing vaccine concerns and enabling parents to continue their child's vaccination.

## Data Availability

The data are not publicly available due to them containing information that could compromise research participants’ consent. Data and materials (i.e. the PACV-Viet) are however available from the authors upon reasonable request. Please contact Dr. Bao Quy Quoc Truong (tqqbao@huemed-univ.edu.vn) for any requests.

## Abbreviations

COVID-19: Coronavirus Disease 2019
PACV: The Parent Attitudes About Childhood Vaccines survey tool
EPI: Expanded Programs on Immunisation
ICC: Intra-class Correlation Coefficient
PCA: Principal Component Analysis
EFA: Exploratory Factor Analysis
KMO: Kaiser-Meyer Olkin
VIF: Variance Inflation Factor
PACV-Viet: The Vietnamese version of the Parent Attitudes About Childhood Vaccines survey tool
OR: Odds Ratio
CI: Confidence interval
AOR: Adjusted Odds Ratio
